# Supplementary material for: Association between oral microbiome and five types of respiratory infections: a two-sample Mendelian randomization study in east Asian population
Source: Front Microbiol. 2024 Apr 10;15:1392473. doi: 10.3389/fmicb.2024.1392473 (PMC11039966; doi:10.3389/fmicb.2024.1392473)
Supplement: Supplementary file 1 [file Data_Sheet_1.zip › Supplementary Materials/MR plots of saliva/Tonsillitis/g__Eggerthia/forest.pdf]

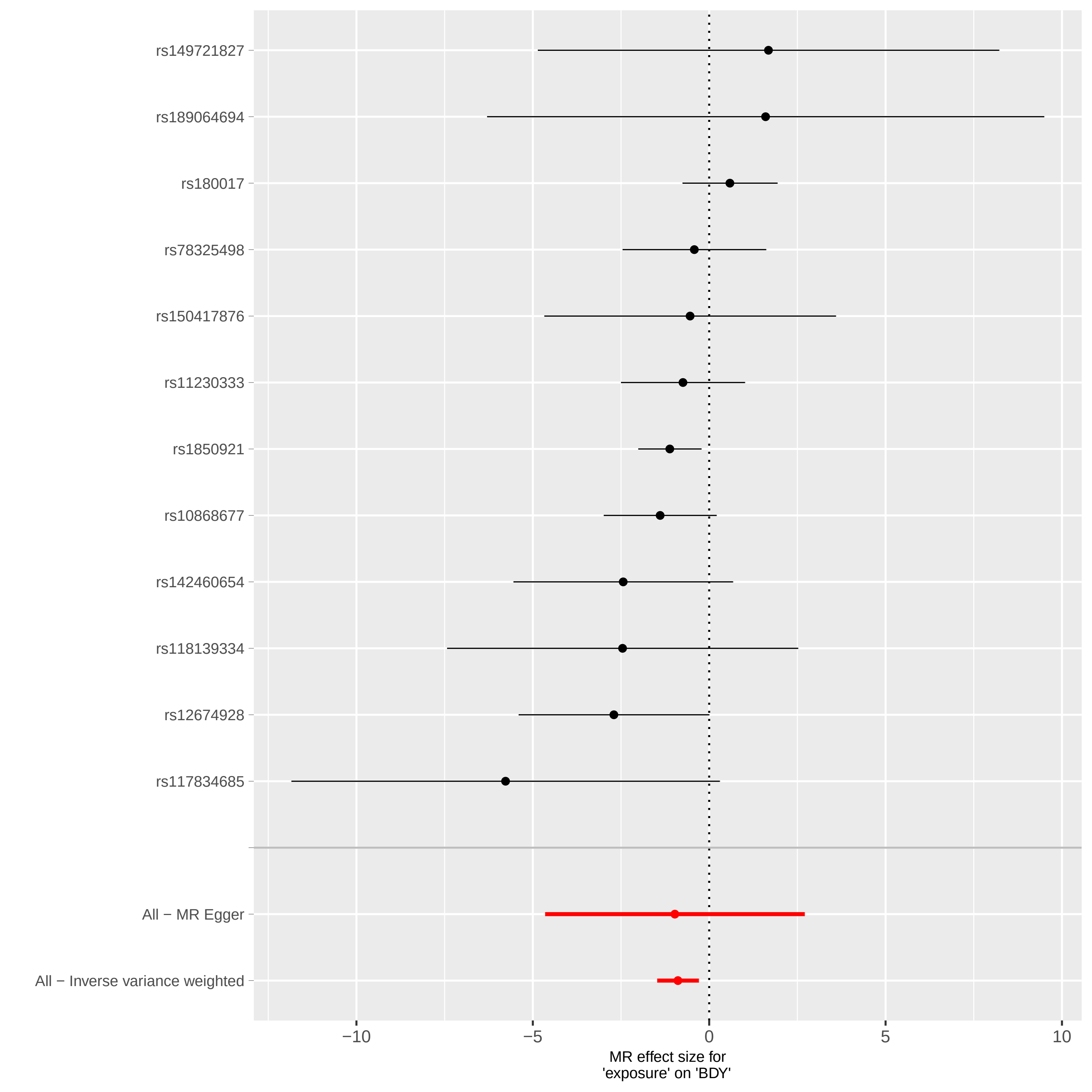
rs149721827
rs189064694
rs180017
rs78325498
rs150417876
rs11230333
rs1850921
rs10868677
rs142460654
rs118139334
rs12674928
rs117834685
All − MR Egger
All − Inverse variance weighted
−10
−5
0
5
10
MR effect size for
'exposure' on 'BDY'
